# Meta-analysis of heritability estimates and genome-wide association for tick-borne haemoparasites in African cattle

**DOI:** 10.3389/fgene.2023.1197160

**Published:** 2023-07-28

**Authors:** Valentina Riggio, Maxime Madder, Michel Labuschagne, Rebecca Callaby, Rongrong Zhao, Appolinaire Djikeng, Josephus Fourie, James G. D. Prendergast, Liam J. Morrison

**Affiliations:** ^1^ The Roslin Institute and Royal (Dick) School of Veterinary Studies, University of Edinburgh, Edinburgh, United Kingdom; ^2^ Clinglobal, Tamarin, Mauritius; ^3^ Centre for Tropical Livestock Genetics and Health (CTLGH), Roslin Institute, University of Edinburgh, Edinburgh, United Kingdom; ^4^ Clinvet, Waverly, NY, United States

**Keywords:** heritability, genome-wide association studies, tick-borne haemoparasites, meta-analysis, African cattle

## Abstract

The control of tick-borne haemoparasites in cattle largely relies on the use of acaricide drugs against the tick vectors, with some vaccination also being used against selected pathogens. These interventions can be difficult in Africa, where accessibility and cost of vaccines can be issues, and the increasing resistance of tick vectors to the widely used acaricides is a complication to disease control. A potential complementary control strategy could be the exploitation of any natural host genetic resistance to the pathogens. However, there are currently very few estimates of the extent of host resistance to tick-borne haemoparasites, and a significant contributing factor to this knowledge gap is likely to be the difficulty of collecting appropriate samples and data in the smallholder systems that predominate livestock production in low- and middle-income countries, particularly at scale. In this study, we have estimated the heritability for the presence/absence of several important haemoparasite species (including *Anaplasma marginale*, *Babesia bigemina*, *Babesia bovis*, and *Ehrlichia ruminantium*), as well as for relevant traits such as body weight and body condition score (BCS), in 1,694 cattle from four African countries (Burkina Faso, Ghana, Nigeria, and Tanzania). Heritability estimates within countries were mostly not significant, ranging from 0.05 to 0.84 across traits and countries, with standard errors between 0.07 and 0.91. However, the weighted mean of heritability estimates was moderate and significant for body weight and BCS (0.40 and 0.49, respectively), with significant heritabilities also observed for the presence of *A. marginale* (0.16) and *E. ruminantium* (0.19)*.* In a meta-analysis of genome-wide association studies (GWAS) for these traits, two peaks were identified as reaching the suggestive significance threshold (*p* < 1.91 × 10^−7^ and *p* < 1.89 × 10^−7^, respectively): one on chromosome 24 for BCS and one on chromosome 8 for the *E. ruminantium* infection status*.* These findings indicate that there is likely to be a genetic basis that contributes to pathogen presence/absence for tick-borne haemoparasite species, which could potentially be exploited to improve cattle resistance in Africa to the economically important diseases caused by these pathogens.

## 1 Introduction

In Africa, livestock forms a critical part of the livelihoods of most rural households, with approximately 162 million low-income livestock keepers (Kruska et al., 2003). In most African countries, livestock is managed under small- to large-scale systems, with small-scale production systems predominating and including pastoral, agro-pastoral, and mixed smallholder farming (Ibeagha-Awemu et al., 2019). They represent approximately 70% of livestock productivity and serve as household assets with multiple livelihood functions, providing food, income, and important non-market services (Ruto et al., 2008). These small-scale systems are characterised by small animal population sizes and low inputs and outputs, which make it difficult to set up programmes aiming at increasing livestock productivity for food production and income generation (Ibeagha-Awemu et al., 2019). However, given the importance of livestock to the diets and incomes of poor farming households and the predicted increase in demand for livestock products throughout the low- and middle-income world over the next few decades, understanding how livestock fits into these systems and how these systems may evolve in the future is critical (Kruska et al., 2003).

The main factors limiting cattle performance under extensive range management in tropical environments are feed resource availability and quality (Hernández-Castellano et al., 2019) and health-related issues, with ticks being among the most important vectors of disease pathogens in livestock and companion animals (Ghosh et al., 2006). Direct effects of tick infestation on cattle include the consumption of blood, causing anaemia and damage to the skin or hide, with downstream effects resulting in reductions in fertility, body weight, and milk production, as well as toxicoses, paralysis, and mortality (Jongejan and Uilenberg, 2004). The economic losses due to ticks and tick-borne diseases (TBDs) have been estimated at more than 3 billion dollars per year in Brazil alone (Grisi et al., 2014). No estimates of economic losses were found for the African continent; however, in Tanzania alone, for example, the total annual national loss due to TBDs was estimated to be 364 million dollars, including the estimated mortality of 1.3 million cattle, mainly attributed to the tick-borne disease East Coast fever, caused by *Theileria parva* (Kivaria, 2006).

The economically most important TBDs of cattle on a global scale are bovine babesiosis, caused by protozoa of the genus *Babesia* (*Babesia bovis* and *Babesia bigemina*), bovine anaplasmosis (*Anaplasma marginale*), bovine theileriosis, caused by *Theileria annulata*, East Coast fever, caused by *Theileria parva*, and ehrlichiosis or heartwater of cattle (*Ehrlichia ruminantium*) (Uilenberg, 1995). Both babesiosis and anaplasmosis can cause severe anaemia, while heartwater, especially in its acute form, can cause a sudden high fever, loss of appetite, depression, and breathing problems. All these diseases have a significant negative economic impact on livestock in tropical countries, due to the expense of vector control, treatment of the disease, effect on animal productivity, and the death of susceptible animals (Mukhebi et al., 1999; Kocan et al., 2003).

In addition to the relatively limited use of live vaccines against a small number of tick-borne pathogens, the strategy most commonly adopted to control tick-borne parasitic diseases is the application of acaricides. The widespread and large-scale use of these acaricides has increased the incidence of acaricide-resistant ticks and exacerbated the occurrence of environmental and food contamination (Parizi et al., 2009; Abbas et al., 2014; Githaka et al., 2022). This has led to an economic and social demand for alternative approaches to reduce tick infestation and thereby enhance the contribution of cattle to the world economy. Natural immunity to the tick vector, which is observed in cattle in environments where ticks are endemic, shows promise for genetic tick control strategies that can reduce expenditure on acaricides and other chemical control methods (Mapholi et al., 2014). Across Africa, it has been common to crossbreed indicine (*Bos taurus indicus*) with taurine (*Bos taurus taurus*) cattle (Jonsson et al., 2008; Cardoso et al., 2021), one reason being that indicine animals are known to be more resistant to ticks as well as TBDs (Silva et al., 2002; Wragg et al., 2022), and this has resulted in generally admixed populations across the continent (Gebrehiwot et al., 2020).

There is precedent for genetic tolerance to vector-borne pathogens proving tractable and potentially applicable as a practical aid in disease control in cattle (Bahbahani and Hanotte, 2015). A very well-defined example is trypanosomiasis, caused by tsetse fly-transmitted protozoan parasites of the *Trypanosoma* genus, where the tolerance of some African taurine breeds has been long recognised and characterised, and loci underpinning this trait have been identified (Murray et al., 1984; Hanotte et al., 2003; Kim et al., 2020). With respect to TBDs, natural tolerance among certain African cattle populations to the *Theileria parva* pathogen has been known for some time. *T. parva* infection is responsible for East Coast fever (ECF), a TBD that causes significant mortality in infected, susceptible cattle in East and Southern Africa. Ndungu et al. (2005) demonstrated that East African Shorthorn Zebu (EASZ) originating from ECF endemic areas showed significantly higher tolerance to *T. parva* challenges than exotic breeds and EASZ from non-endemic areas. Likewise, a notable recent study by Wragg et al. (2022) highlighted that certain lineages of Boran cattle also show elevated tolerance to *T. parva* infection and identified a locus associated with tolerance to ECF. The identification of this locus by Wragg et al. (2022) raises the promise of marker-assisted selection for cattle that are less susceptible to infection by *T. parva*. However, in general, only a few studies have reported naturally occurring loci with large effects that confer resistance to disease in livestock (White et al., 2014; Chen et al., 2015; Matika et al., 2019), and studies of natural tolerance to most African cattle pathogens remain limited.

Resistance to diseases is usually a physiologically complex trait that develops over time, with the mechanisms underlying genetic differences in resistance being poorly understood. Traditionally, the genetic control of complex traits in livestock has been based on estimating breeding values from phenotypic and pedigree information, without identifying the genes or gene variants underlying the observed variation (Goddard and Hayes, 2009), which can be a challenge in most African husbandry systems. However, current molecular tools allow animal geneticists to investigate the nature of genetic variation underlying complex traits, without requiring pedigree information, making selection a possibility.

While several genome-wide association studies (GWAS) have already reported quantitative trait loci for resistance to ticks (see Mapholi et al., 2014 for a review) in cattle, very few studies investigating loci underlying variation in resistance to tick-borne haemoparasites are reported in the literature.

Although a variety of arrays are currently available for genotyping in cattle, they are mostly designed for European breeds and are, therefore, biased toward variants common to these breeds. This can make it difficult to map the genetic loci that underlie important traits in African cattle, which are a mixture of African taurine and indicine backgrounds (Gebrehiwot et al., 2020). A recent study demonstrated that the Illumina HD (https://www.illumina.com/ documents/products/datasheets/datasheet_bovine HD.pdf) and the Axiom Genome-Wide BOS1 (https://www.thermofisher.com/order/catalog/product/901791#/901791) arrays are the best at capturing the diversity across African breeds/populations, even if compared with *Bos t. indicus*-specific arrays (Riggio et al., 2022). Another limitation to performing GWAS on African cattle is the small size of most herds, which limits the power to detect significant associations. In this case, a meta-analysis, combining results across independent studies, could help boost statistical power (Munafò and Flint, 2004). This study aimed to investigate the potential of breeding for cattle resistant or tolerant to particular TBDs by characterising the heritability of relevant traits and investigating the potential of identifying loci underlying the variation in the presence of tick-borne haemoparasites, as well as important traits such as body weight and body condition score (BCS), in African cattle from four different countries (Burkina Faso, Ghana, Nigeria, and Tanzania).

## 2 Materials and methods

### 2.1 Population

**FIGURE 1:**
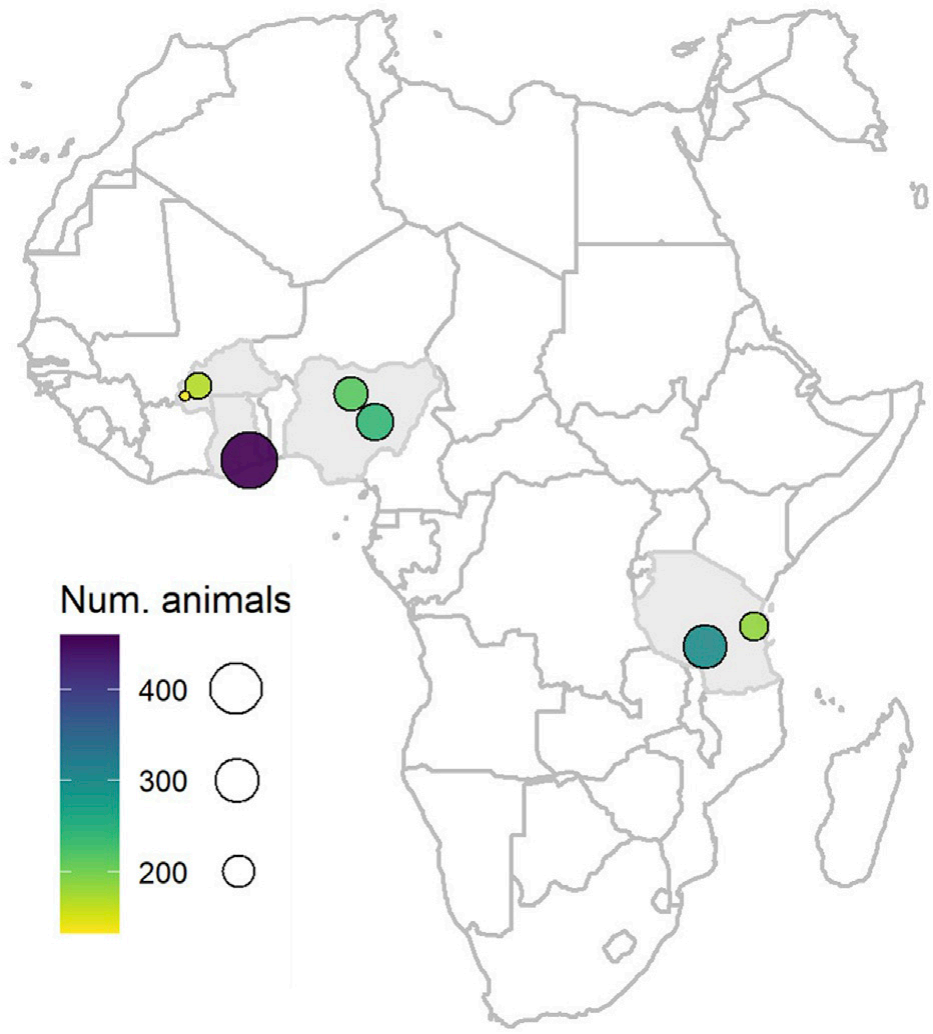
Map showing the major sampling locations across the four African countries (Burkina Faso, Ghana, Nigeria, and Tanzania). Sampling locations are represented by circles, with size and colour changing according to the numbers of animals.

The population consisted of 1,694 one- to two-year-old cattle across four African countries (Burkina Faso, n = 300; Ghana, n = 459; Nigeria, n = 454; and Tanzania, n = 481), which is a subset of the 1,740 cattle used in Riggio et al. (2022). Sampling sites within the country consisted of smallholder farmer settlements with herds that were mainly sedentary, within at least two districts per country, chosen taking into consideration high cattle density and an expected or previously recorded high prevalence of ticks and TBDs. These farmers were considered to belong to communities of limited-resource stockbreeders. Up to four visits per sampling site were conducted over a period of 12 months (between August 2016 and August 2017) in order to cover both the rainy and dry seasons (see Heylen et al., 2023a; Heylen et al., 2023b for more details). However, in the current study, only one record per animal was available. Figure 1 shows the major sampling locations across the four African countries. The map was drawn using the maps package in R (https://cran.r-project.org/web/packages/maps/index.html).

### 2.2 Phenotypic measurements

Body weight was estimated using a Rondo tape according to the manufacturer’s recommendations (Agrihealth Rondo). It is based on the measurement of thoracic girth, which is reported to be highly correlated with body weight in cattle (Heinrichs et al., 2007; Swali et al., 2008). Body condition scoring (BCS) was also conducted for these animals. BCS was based on a subjective classification scale of nine points, from extremely thin (i.e., score 1) to extremely fat (i.e., score 9), as reported by Arango et al. (2002).

Blood samples (approximately 125 μL) were collected from the middle ear vein using a sterile lancet and capillary tube. Samples were then applied onto Whatman^®^ FTA^®^ cards, air-dried, labelled appropriately, and packed in foil pouches with a silica gel, prior to DNA isolation. DNA was isolated from 2 × 5 mm diameter blood-soaked FTA card punches using the MagMAX DNA Multi-Sample Ultra Kit according to the manufacturer’s recommendations. DNA isolation was performed using the KingFisher 96-flex instrument, processing 92 samples per run and including extraction controls for each run. Eluted DNA was collected in a final volume of 75 µL and used for downstream processing. Testing was performed to assess the absence/presence (i.e., 0/1) of anaplasmosis, babesiosis, theileriosis, and ehrlichiosis. The inhibitor tolerant and highly processive SsoAdvanced Universal Probes Supermix DNA polymerase master mix (Bio-Rad) was used in all PCR assay development and final detection assays. Published assay primers and probes (Kim et al., 2007; Decaro et al., 2008; Steyn et al., 2008) were evaluated in a single target and multiplex environment using sequence-verified linear synthetic DNA templates to determine the limit of detection (LOD) for each assay in the presence of 10 ng bovine DNA. Final multiplex PCR assay combinations were based on results that exhibited the same LOD in both single and multiplex PCR setups (data not shown). The Hamilton Nimbus robotic liquid handling system was used for mastermix and template addition to a 384-well PCR plate. All plates contained positive control samples (synthetic DNA representing 100 copies of each target region per reaction), negative control (10 ng bovine DNA from a donor animal to exclude host-related amplification), and extraction control (to exclude extraction kit-related amplification). A measure of 5 µL of template DNA was used in a 15 µL final PCR reaction and was subjected to thermal cycling consisting of initial denaturation at 98°C for 3 min, followed by 45 cycles of 95°C for 15 s and 60°C for 30 s, making use of the QuantStudio6 qPCR system. Data captured during thermal cycling were analysed using QuantStudio Real-Time PCR software v1.2. Samples exhibiting the correct amplification profiles were called as detected (i.e., presence = 1), whereas the rest were called not detected (i.e., absence = 0).

### 2.3 Genotypic data

Animals were genotyped using the Illumina HD genotyping array. The SNP genotype data were subjected to quality control (QC) measures, as previously described in Riggio et al. (2022). QC was conducted per country, and the SNPs with a minor-allele frequency (MAF) < 0.01 or a call rate <90% were removed. Furthermore, markers on the sex chromosomes were removed from the analysis. After QC, 585,754 SNPs were retained for the Burkina Faso population, 594,060 for Ghana, 586,851 for Nigeria, and 595,683 for Tanzania and used for variance component estimation analyses. The positions of SNP markers were relative to the ARS-UCD1.2 bovine genome assembly (Rosen et al., 2020). Genome-wide association analyses were subsequently performed using 10,282,187 variants previously imputed by Riggio et al. (2022). Only variants with an imputation accuracy *r* ^2^ (as obtained from Minimac4 software, https://genome.sph.umich.edu/wiki/Minimac4) >0.85 and a MAF >0.05 were considered for the within-population GWAS (i.e., out of the 10,282,187 imputed genotypes, 5,460,147 were retained for Burkina Faso, 5,477,083 for Ghana, 5,474,804 for Nigeria, and 5,464,291 for Tanzania).

### 2.4 Statistical analyses

Descriptive statistics were calculated for body weight and BCS for each country, whereas prevalence was calculated for the haemoparasites and defined as the percentage of animals that were positive by qPCR at any time during the period considered in this study.

Initial data exploration and model development were conducted in SAS version 9.4, using the PROC GLM (SAS Institute Inc., Cary, NC). Variance components for all traits were estimated within the country using ASReml 4 (Gilmour et al., 2015), fitting an animal model with a logit function for the binary traits (i.e., the haemoparasite traits). The fixed effects were: sex (two levels, male and female), district (two levels), with visit number, and the first five genetic principal components (PCs) as covariates. The PCs were included as a substitute for the effects of breed, to account for population structure. The animal was fitted as a random effect, and the relationship matrix (GRM, **G**) was calculated using VanRaden’s method 2 (VanRaden, 2008), with all SNPs from the HD array. The rma function in the metafor package (Viechtbauer, 2010) in R was used to obtain the weighted mean of heritability estimates (h^2^_m_) across the four African countries. In addition, Q statistics (Cochran, 1954) were used to quantify the degree of heterogeneity among countries for each trait.

The GWAS analyses for each country were performed using GEMMA (Zhou and Stephens, 2012) using the imputed genotypes. The same fixed (i.e., sex, district, visit number, and the first five genetic PCs) and random (i.e., animal) effects used for the variance component analysis were fitted, assuming a model where the vectors of random effects, **u**, and errors, **ϵ**, follow multivariate normal (MVN) distributions given by **u** ∼ MVN (0,V_G_ **G**) and **ϵ** ∼ MVN (0,V_E_ **I**), where V_G_ and V_E_ are the genetic variances associated with **G** and environmental variance, respectively. GEMMA provides a regression coefficient for each SNP, and their statistical significance was assessed using a Wald test. A Bonferroni correction was used to account for multiple testing and identify the *p*-values for genome-wide (*p* < 0.05) and suggestive (i.e., one false positive per genome scan) significance thresholds. The within-country analyses were followed by a meta-analysis using a weighted Z-score model as implemented in the METAL software (Willer et al., 2010). The weighted Z-score model used *p*-values, directions of effect estimates, and weights in individual GWAS based on the sample size to compute a Z-score. The heterogeneity of the effect sizes across countries was evaluated using Cochran’s Q-test (Cochran, 1954) as implemented in the METAL software (Willer et al., 2010). Only common SNPs across countries were considered.

Genes overlapping the regions of interest were identified using the biomaRt package in R (Durinck et al., 2009). Variants in the regions were those identified in cohorts of 92 African cattle (Dutta et al., 2020), and the linkage disequilibrium (LD) between the target variants and other variants was calculated using PLINK 1.9 (Purcell et al., 2007). The Manhattan plots and tracks were made using the ggplot2 (Wickham, 2016), dplyr (Wickham et al., 2023), and Gviz (Hahne and Ivanek, 2016) packages in R.

## 3 Results

To estimate the heritability of the presence of tick-borne haemoparasites, body weight, and body condition score in African cattle, we analysed four independent datasets from Burkina Faso, Ghana, Nigeria, and Tanzania, for a total of 1,694 cattle. Heritability estimates were then used to calculate the weighted mean of heritability estimates (h^2^_m_ **)** across countries. The same data were also used to carry out GWAS to identify potential loci underlying genetic variation in the traits of interest, with a meta-analysis across countries being conducted.

Means, standard deviations, coefficients of variation, and ranges of the continuous traits considered in this study (i.e., body weight and BCS) are presented per country in Table 1. Body weight values over three standard deviations from the mean were removed. Although there is some variation across countries for body weight, coefficients of variation (CVs) are similar for all countries, ranging between 30 and 39, with Nigeria having the highest value. This is confirmed by the histogram in Supplementary Figure S1, which shows higher variability in the body weight distribution for Nigeria. More variability was observed for the BCS, with CVs ranging between 18 for Ghana and 34 for Burkina Faso (see also Supplementary Figure S2, showing the distribution of BCS categories across the four countries).

**TABLE 1:** Descriptive statistics of body weight and body condition score per country.

	Body weight (kg)			Body condition score (1–9)		
	Mean ± SD	CV (%)	Min–max	Mean ± SD	CV (%)	Min–max
Burkina Faso	193.2 ± 62.4	32	70.0–378.0	4.09 ± 1.38	34	2–9
Ghana	96.4 ± 29.3	30	70.0–220.0	5.37 ± 0.97	18	3–8
Nigeria	128.0 ± 49.7	39	46.0–339.0	4.41 ± 0.98	22	1–7
Tanzania	126.8 ± 43.4	34	50.0–290.0	5.58 ± 1.09	20	3–8

Table 2 shows the prevalence of different haemoparasites as assayed by species-specific qPCR across the four countries. The prevalence of *Theileria* is not presented as it was not detected in any sample. The highest prevalence was observed for *A. marginale* across all countries (ranging between 43% and 97%), with Ghana presenting the highest prevalence. In general, prevalence was lower in Burkina Faso than in other countries for all parasites considered. This might have an impact on the estimation of the heritabilities and analyses of GWAS, as there is insufficient information to generate reliable results. Similarly, the analyses for *A. marginale* in Ghana may be affected by the very high prevalence.

**TABLE 2:** Prevalence (%) of haemoparasitic infections per country. The number of individuals with information used to calculate the prevalence is also reported.

	Burkina Faso (n = 218)	Ghana (n = 459)	Nigeria (n = 331)	Tanzania (n = 481)
*Anaplasma marginale*	43	97	47	72
*Babesia bigemina*	1	20	22	29
*Babesia bovis*	1	9	1	9
*Ehrlichia ruminantium*	2	12	5	6

Table 3 shows the heritability (h^2^) estimates for body weight and BCS as well as the haemoparasite traits for each country. For most traits, the standard errors of the heritability estimates were large, making the estimated h^2^ not significant. While these estimates are not significant and there is little consistency across countries, there is evidence of genetic variation. These results are probably a reflection of the challenges of collecting consistent phenotypes in the settings concerned, as well as the limitations associated with issues such as small herd sizes.

**TABLE 3:** Heritability (h^2^) estimates (±s.e.) for body weight, BCS, and haemoparasite traits per country. Significant estimates are also shown (*).

	Burkina Faso	Ghana	Nigeria	Tanzania
Body weight	0.67 ± 0.39	0.14 ± 0.17	0.54 ± 0.13^*^	0.39 ± 0.19^*^
Body condition score	0.25 ± 0.25	0.60 ± 0.15^*^	0.25 ± 0.14	0.84 ± 0.18^*^
*Anaplasma marginale*	0.10 ± 0.13	0.29 ± 0.21	0.18 ± 0.09^*^	0.15 ± 0.07^*^
*Babesia bigemina*	0.40 ± 0.35	0.07 ± 0.11	0.15 ± 0.13	0.09 ± 0.08
*Babesia bovis*	0.22 ± 0.91	0.12 ± 0.17	0.20 ± 0.59	0.08 ± 0.18
*Ehrlichia ruminantium*	0.42 ± 0.32	0.05 ± 0.16	0.21 ± 0.23	0.25 ± 0.14

Table 4 presents the weighted mean of heritability estimates (h^2^_m_) and Q statistics for each trait. The h^2^_m_ was moderate and significant (*p* < 0.05) for both body weight and BCS (0.40 and 0.49, respectively), with significant Q statistics for BCS. When looking at the haemoparasite traits, although the h^2^_m_ estimates are low, ranging between 0.10 for *B. bigemina* and 0.19 for *E. ruminantium*, the estimates for *A. marginale* and *E. ruminantium* are both significant, suggesting a genetic component to the presence of these parasites in African cattle.

**TABLE 4:** Weighted means of heritability estimates (h^2^_m_) and their Q statistics (Q) for body weight, BCS, and haemoparasite traits across countries.

	h^2^_m_ ± s.e	Q statistics	Q *p*-value
Body weight	0.40 ± 0.11^a^	3.98	0.26
Body condition score	0.49 ± 0.14^a^	8.18	0.04
*Anaplasma marginale*	0.16 ± 0.05^a^	0.67	0.88
*Babesia bigemina*	0.10 ± 0.06	0.97	0.81
*Babesia bovis*	0.11 ± 0.12	0.07	0.99
*Ehrlichia ruminantium*	0.19 ± 0.09^a^	1.47	0.69

^a^ (*p* < 0.05).

**FIGURE 2:**
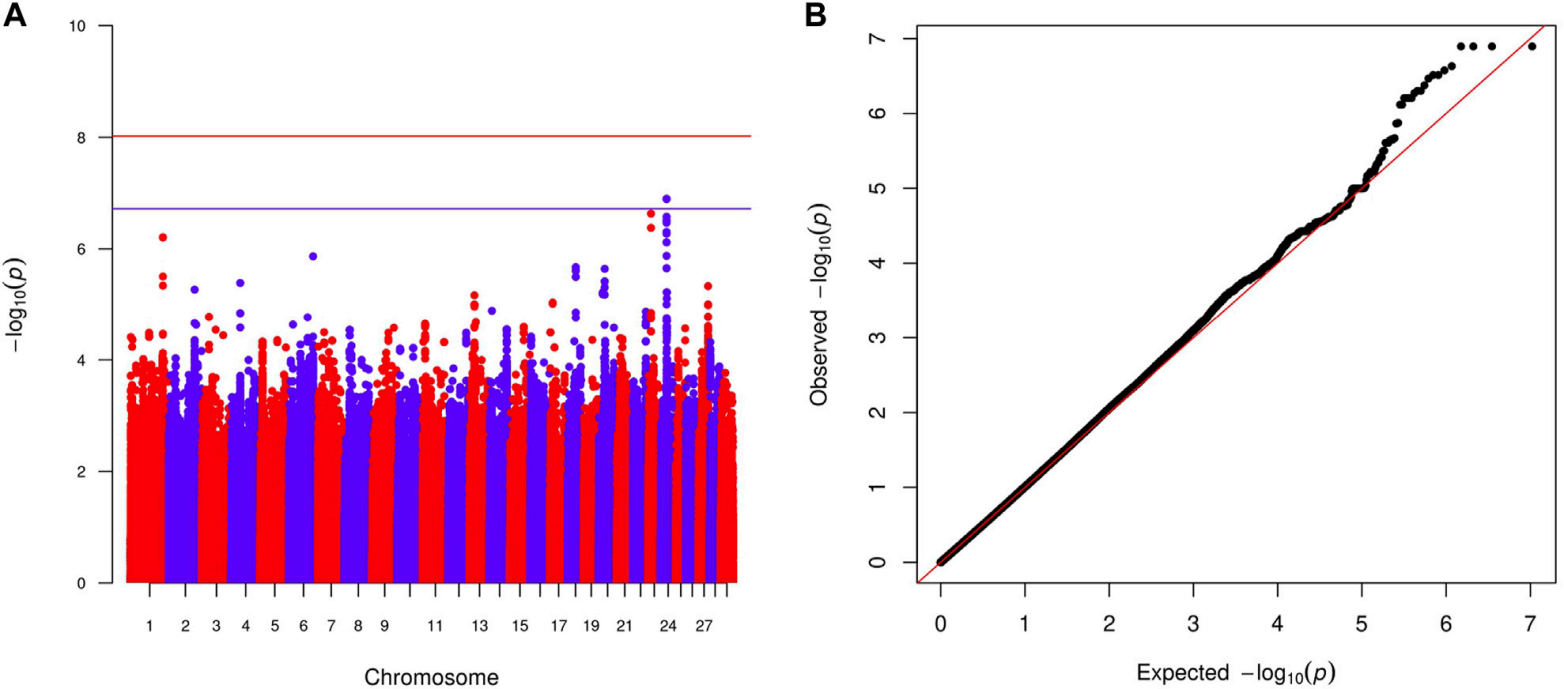
Manhattan plot **(A)** displaying the meta-analysis results (-log_10_ (*p*) of the corresponding *p*-values) and Q–Q plot **(B)** of observed *p*-values against the expected *p*-values for the body condition score. Genome-wide *p* < 0.05 (red line; *p* < 9.52 × 10^−9^ corresponding to -log_10_ (*p*) of 8.02) and suggestive (blue line; *p* < 1.91 × 10^−7^ corresponding to -log_10_ (*p*) of 6.72) significance thresholds are also shown.

**FIGURE 3:**
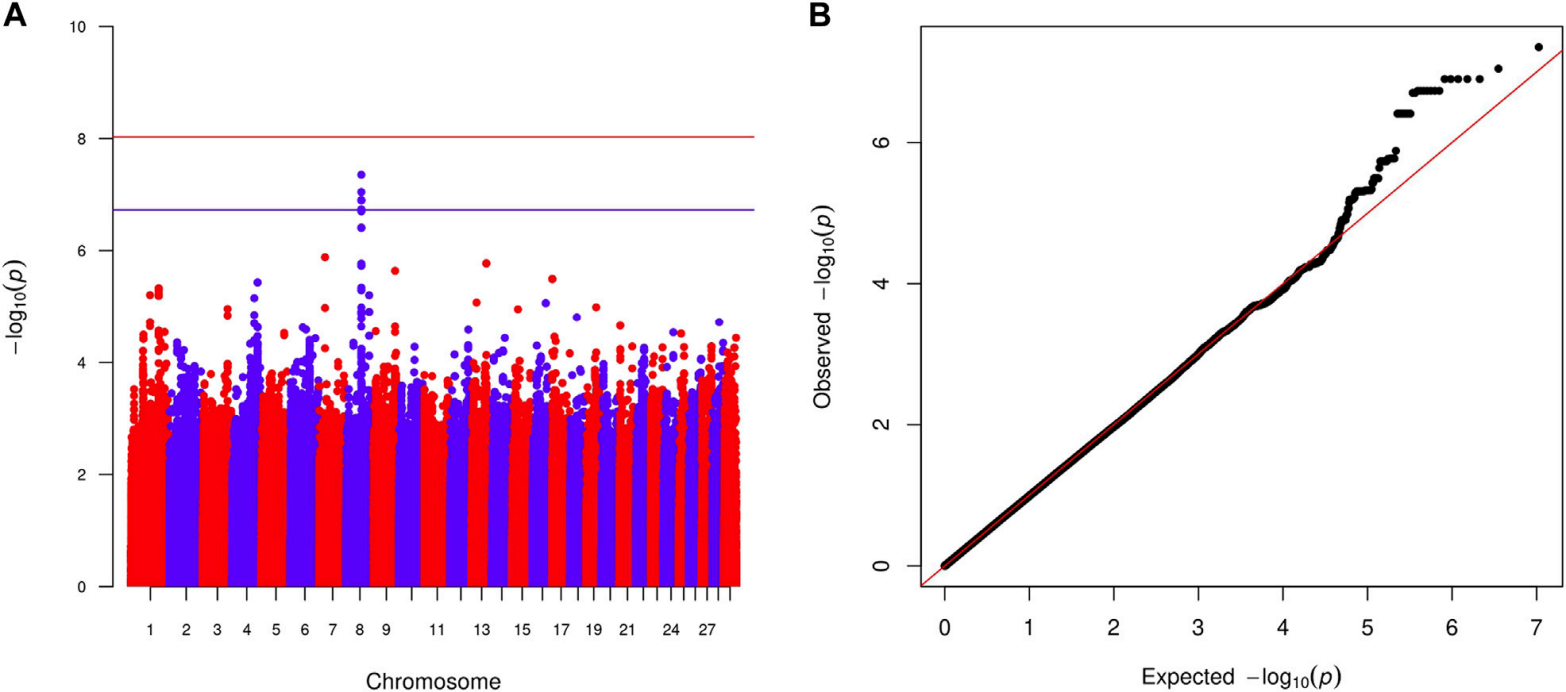
Manhattan plot **(A)** displaying the meta-analysis results (-log_10_ (*p*) of the corresponding *p*-values) and Q–Q plot **(B)** of observed *p*-values against the expected *p*-values for the *Ehrlichia ruminantium* infection status. Genome-wide *p* < 0.05 (red line; *p* < 9.42 × 10^−9^ corresponding to -log_10_ (*p*) of 8.03) and suggestive (blue line; *p* < 1.89 × 10^−7^ corresponding to -log_10_ (*p*) of 6.73) significance thresholds are also shown.

**FIGURE 4:**
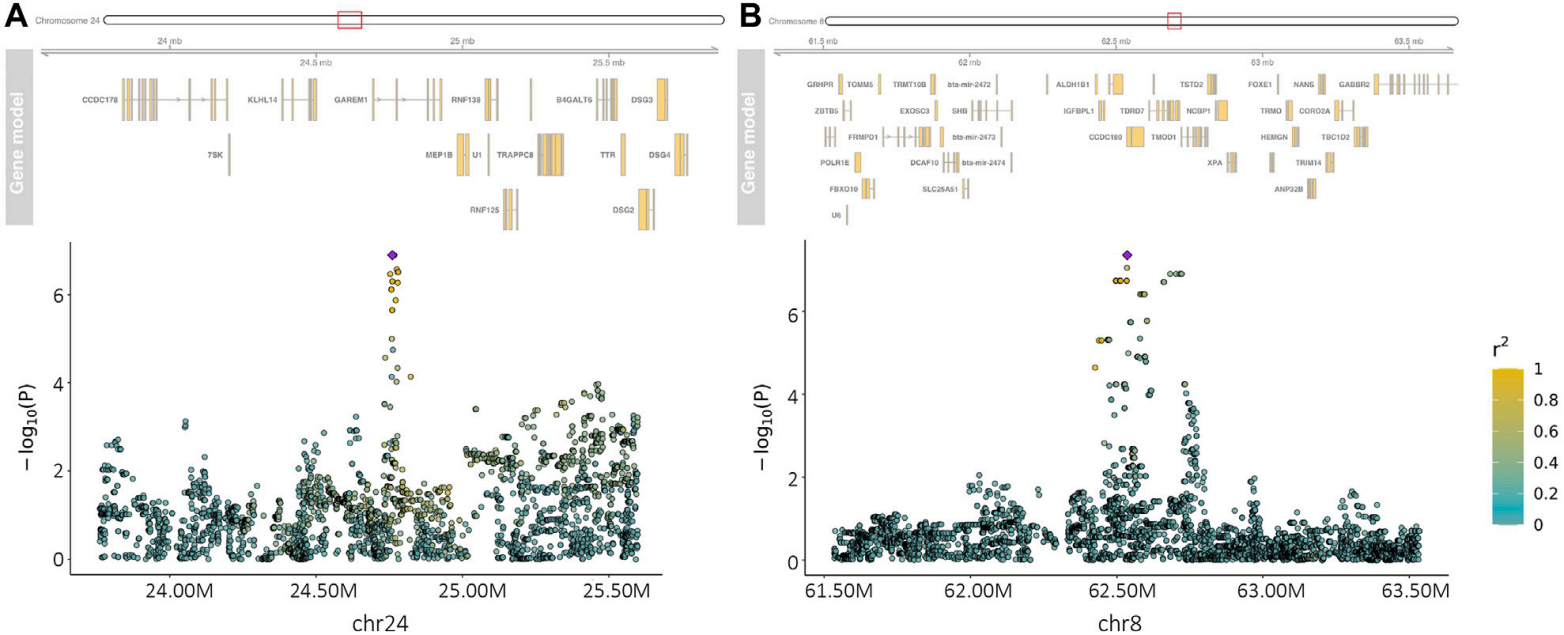
Manhattan plot of the regions of interest on chromosome 24 for the body condition score **(A)** and on chromosome 8 for the *Ehrlichia ruminantium* infection status **(B)**. The purple diamond in the plots represents the variant with the smallest *p*-value in the areas of interest. Other variants in the area are coloured according to their LD (*r* ^2^) with the target variant.

Within-country GWAS analyses did not generally show any regions associated with the traits of interest, reflecting the low sample numbers. To overcome this limitation, we performed meta-analyses across countries for the two quantitative and four haemoparasite phenotypes. When the prevalence of a phenotype was ≥0.05 or ≤0.95 within a country, the corresponding results were excluded from these meta-analyses. Across these six phenotypes, two peaks reached suggestive significance (*p* < 1.91 × 10^−7^ and *p* < 1.89 × 10^−7^, respectively). The first peak (lead variant: chr24:24760549:A:G; *p* = 1.2 × 10^−7^) was observed for the body condition score on chromosome 24 (Figure 2), with this peak overlapping the *GAREM1* gene (Figure 4A) that has previously been linked to body mass in mice and humans (Nishino et al., 2022). The second peak (lead variant: chr8:62534748:T:C; *p* = 4.5 × 10^−8^) was identified for the *E. ruminantium* infection status on chromosome 8 (Figure 3), which overlaps seven genes (Figure 4B). Of these, the *Tudor domain*-*containing protein 7* (*TDRD7*) gene has previously been associated with immune responses.

## 4 Discussion

Selection programmes for the improvement of host resistance against ticks and tick-borne diseases could be considered an important strategy for reducing the expenses associated with treatment and prophylaxis management. However, the implementation of genetic improvement programmes has always been difficult in low- and middle-income country settings, due to many factors, including cost, lack of or poor infrastructure, and small herds, which hinder the estimation of population-specific parameters (i.e., heritability and genetic correlations) for traits of economic importance. Moreover, traditionally, the implementation of a breeding programme required an accurate pedigree. In smallholder properties in tropical and subtropical environments, there is often no pedigree recording and no phenotype data recording, rendering conventional breeding practices impossible to implement. Current molecular tools allow for investigating the genetics of complex traits without requiring pedigree information, making selection based on the genome a possibility. One of the key shortcomings of using these technologies in low- and middle-income countries is the cost associated with them. Genotyping arrays are currently the most commonly used tool in GWAS. However, the current commercial genotyping arrays are often biased toward variants common to European breeds, and we have recently shown that they poorly tag variants segregating in indicine breeds, with implications for performing GWAS in African breeds (Riggio et al., 2022). Further limitations associated with performing GWAS in African cattle are the generally small sample size and large variability in farming practices as well as the extensive admixture of these populations (Gebrehiwot et al., 2020). Combining results across independent studies via meta-analysis could help partly overcome these issues, boosting the power to detect significant associations.

To investigate the genetic architecture of the presence of haemoparasites, as well as body weight and BCS, in African cattle, we estimated heritabilities and carried out GWAS using 1,694 cattle from four different countries (Burkina Faso, Ghana, Nigeria, and Tanzania) with genotypes imputed from HD to the whole-genome level, both independently and via meta-analysis. We have shown evidence of genetic variation for most traits, with the weighted mean of heritability estimates being moderate for body weight and BCS and low for haemoparasites, and we have identified two peaks reaching the suggestive significance threshold (i.e., one false positive per genome scan) in the meta-analysis*.* We could not include *Theileria parva* in the analysis as we did not detect any infections in our sample set. This was expected for Ghana, Burkina Faso, and Nigeria but was surprising for Tanzania, where both the tick vector and *Theileria parva* are endemic (Kerario et al., 2017; Allan et al., 2021). The explanation for the lack of detection is either lack of sensitivity based on the substrate (DNA from FTA cards is less effective than DNA purified from blood (Hailemariam et al., 2017)), the assay used (only primers suitable for use in qPCR were considered for this study, which may have compromised sensitivity), or lack of detectable infection in the target animals (it is probable that any infected animals would be carriers, meaning low parasite numbers). However, the lack of detectable infections meant that we were not able to assess heritability for this important pathogen, although numbers and, therefore, power would have been very low in any case given the restriction to one country.

Although heritability estimates for body weight and BCS within the country were mostly not significant, the weighted means of heritability estimates (h^2^_m_) were moderate and significant (i.e., 0.40 for body weight and 0.49 for BCS). This estimate of the heritability of body weight is consistent with that previously estimated within European cattle, where values generally fall between 0.4 and 0.7 (Mehtiö et al., 2021). Likewise, heritability estimates for BCS in European cattle breeds have generally fallen within the range of 0.2–0.5 (Bastin and Gengler, 2013). Consequently, our heritability estimates for these traits are consistent with previous findings.

Estimates for haemoparasites were lower, ranging between 0.10 and 0.19. Low heritability estimates for the infection levels of *B. bigemina* and *B. bovis* (i.e., 0.09 and 0.1, respectively) were also previously reported by Romero (2021). Although we use a different definition of the trait, as we are only considering the presence/absence of the pathogen, both results seem to be consistent in showing a high environmental influence on such traits. Romero (2021) also reported a heritability estimate of 0.09 for the infection level of *A. marginale.*

Previous studies have identified a region on chromosome 5 associated with BCS in tropical cattle (Porto-Neto et al., 2014), but there was little evidence of an association with this locus in our analyses. However, the authors in that study visually assessed body condition at an average of 30 months of age and subjectively scored at 1/3rd score increments from 1 to 5 and subsequently converted it to a continuous 15-point scale (Porto-Neto et al., 2014), whereas we used a classification scale of nine points. In this study, we identified a region reaching the suggestive significance level on chromosome 24, which overlaps the *GAREM1* gene. In a study on body mass in humans and mice, Nishino et al. (2022) found that *GAREM1* is required for normal growth and for maintaining average body size in these species. This gene is consequently a potentially interesting candidate for follow-up work further exploring its potential link to BCS in African cattle.

Considerable work has been carried out to address the genetic control of resistance to ticks [see Mapholi et al. (2014) for a review]. To the best of our knowledge, few studies have been published so far for TBDs (Romero, 2021; Wragg et al., 2022). Comparisons across studies are not easy, given the complexity of the traits and the differences among populations and in tick/pathogen prevalence, as well as the approaches used. Moreover, in contrast to association studies in humans, where in general largely unrelated individuals are used, livestock populations are often characterised by high levels of relatedness (i.e., closely related animals with a complex population structure) and an *a priori* unbalanced distribution of allele frequencies, which is likely to inflate the rate of false-positive associations between the traits and the markers, making true associations harder to detect. However, our meta-analysis showed evidence of a peak on chromosome 8 for the *E. ruminantium* infection status. This region overlaps several genes, though *TDRD7* is potentially the strongest candidate for being linked to the infection status. Given that *E. ruminantium* is an intracellular bacterium, this gene could be relevant as it is an important factor involved in cellular responses to viral infection (Thakur et al., 2019; Subramanian et al., 2020; Forst et al., 2022). A previous study from Romero (2021) in a similar number of South American cattle, using predominantly low-density arrays, prioritised several regions across the genome potentially affecting the infection level of *B. bigemina* (chromosomes 5, 7, 10, 20, and 27) and *A. marginale* (chromosomes 2, 5, 8, 10, 13, 15, 17, 20, 24, and 29). However, none of these regions were significant in our analyses.

In conclusion, in this study, we aimed at estimating heritabilities and identifying loci underlying genetic variations for important haemoparasites, as well as body weight and body condition score, in African cattle. Despite the relatively modest heritability estimates in the within-country analysis, our meta-analysis results have indicated the possibility of improving these traits. Moreover, our meta-analyses have identified two regions associated with body condition score and presence/absence of *E. ruminantium*, which should be further explored. These data suggest that future larger-scale studies to explore the genetic basis of resistance/tolerance to tick-borne pathogens have merit, as improving the resolution by increasing animal numbers has the potential to identify genetic traits that may contribute in the future to reducing the disease burdens on smallholder farmers in Africa.

## Data Availability

Raw Illumina HD genotypes (i.e., 777,962 SNPs) mapped to the bovine UMD3.1 genome assembly for the 1,694 cattle used in this study have been previously uploaded on Zenodo with https://doi.org/10.5281/zenodo.6791394. The phenotype datasets generated during the study are not publicly available due to Contract Research Organization agreements (data will be stored in the archives of Clinglobal, Mauritius), but are available from the corresponding author on reasonable request.
