# Supplementary material for: Meta-analysis of heritability estimates and genome-wide association for tick-borne haemoparasites in African cattle
Source: Front Genet. 2023 Jul 28;14:1197160. doi: 10.3389/fgene.2023.1197160 (PMC10417722; doi:10.3389/fgene.2023.1197160)
Supplement: Supplementary file 1 [file DataSheet1.docx]

**Supplementary Figure 1**

Distribution of the body weight data per country

**
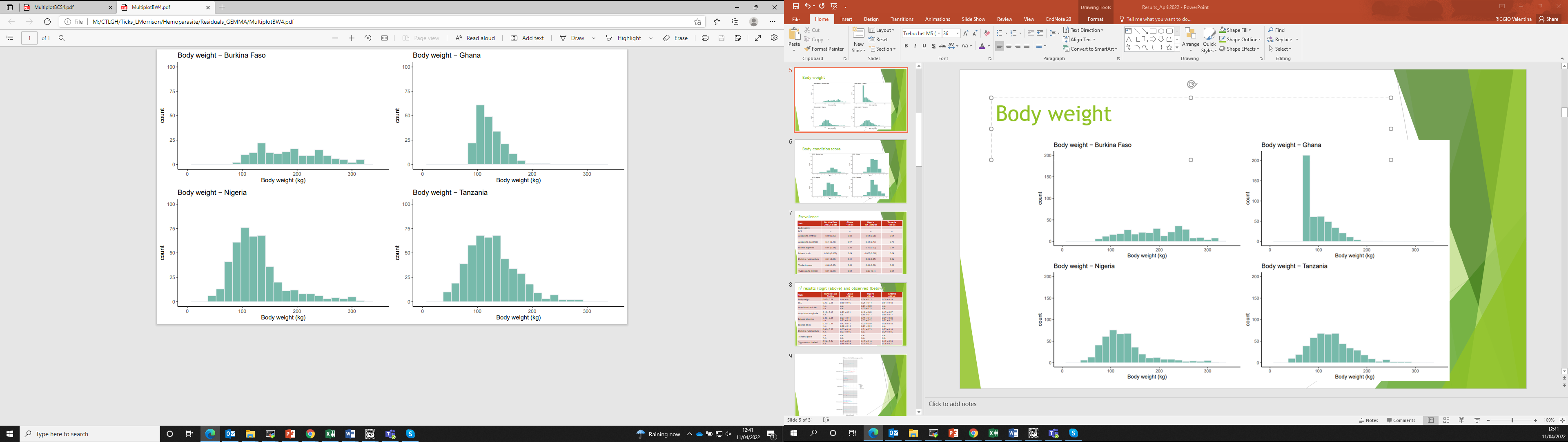
**

**Supplementary Figure 2**

Distribution of the body condition score (BCS) data per country

**
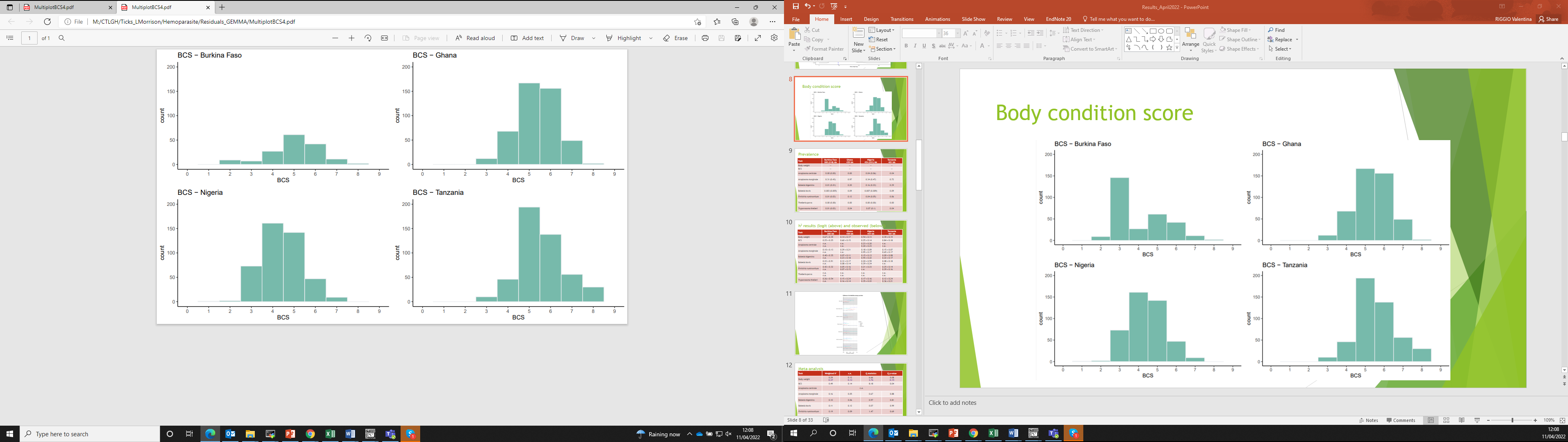
**
